# Detection of cancer‐associated cachexia in lung cancer patients using whole‐body [^18^F]FDG‐PET/CT imaging: A multi‐centre study

**DOI:** 10.1002/jcsm.13571

**Published:** 2024-08-27

**Authors:** Daria Ferrara, Elisabetta M. Abenavoli, Thomas Beyer, Stefan Gruenert, Marcus Hacker, Swen Hesse, Lukas Hofmann, Smilla Pusitz, Michael Rullmann, Osama Sabri, Roberto Sciagrà, Lalith Kumar Shiyam Sundar, Anke Tönjes, Hubert Wirtz, Josef Yu, Armin Frille

**Affiliations:** ^1^ QIMP Team Medical University of Vienna Vienna Austria; ^2^ Division of Nuclear Medicine Azienda Ospedaliero Universitaria Careggi Florence Italy; ^3^ Division of Nuclear Medicine Medical University of Vienna Vienna Austria; ^4^ Department of Nuclear Medicine University Hospital Leipzig Leipzig Germany; ^5^ Department of Respiratory Medicine University Hospital Leipzig Leipzig Germany; ^6^ Department of Endocrinology University Hospital Leipzig Leipzig Germany

**Keywords:** [^18^F]Fluoro‐2‐deoxy‐D‐glucose, Cachexia, Lung cancer, Metabolism, PET/CT

## Abstract

**Background:**

Cancer‐associated cachexia (CAC) is a metabolic syndrome contributing to therapy resistance and mortality in lung cancer patients (LCP). CAC is typically defined using clinical non‐imaging criteria. Given the metabolic underpinnings of CAC and the ability of [^18^F]fluoro‐2‐deoxy‐D‐glucose (FDG)‐positron emission tomography (PET)/computer tomography (CT) to provide quantitative information on glucose turnover, we evaluate the usefulness of whole‐body (WB) PET/CT imaging, as part of the standard diagnostic workup of LCP, to provide additional information on the onset or presence of CAC.

**Methods:**

This multi‐centre study included 345 LCP who underwent WB [^18^F]FDG‐PET/CT imaging for initial clinical staging. A weight loss grading system (WLGS) adjusted to body mass index was used to classify LCP into ‘No CAC’ (WLGS‐0/1 at baseline prior treatment and at first follow‐up: *N* = 158, 51F/107M), ‘Dev CAC’ (WLGS‐0/1 at baseline and WLGS‐3/4 at follow‐up: *N* = 90, 34F/56M), and ‘CAC’ (WLGS‐3/4 at baseline: *N* = 97, 31F/66M). For each CAC category, mean standardized uptake values (SUV) normalized to aorta uptake (<SUV_aorta_>) and CT‐defined volumes were extracted for abdominal and visceral organs, muscles, and adipose‐tissue using automated image segmentation of baseline [^18^F]FDG‐PET/CT images. Imaging and non‐imaging parameters from laboratory tests were compared statistically. A machine‐learning (ML) model was then trained to classify LCP as ‘No CAC’, ‘Dev CAC’, and ‘CAC’ based on their imaging parameters. SHapley Additive exPlanations (SHAP) analysis was employed to identify the key factors contributing to CAC development for each patient.

**Results:**

The three CAC categories displayed multi‐organ differences in <SUV_aorta_>. In all target organs, <SUV_aorta_> was higher in the ‘CAC’ cohort compared with ‘No CAC’ (*P* < 0.01), except for liver and kidneys, where <SUV_aorta_> in ‘CAC’ was reduced by 5%. The ‘Dev CAC’ cohort displayed a small but significant increase in <SUV_aorta_> of pancreas (+4%), skeletal‐muscle (+7%), subcutaneous adipose‐tissue (+11%), and visceral adipose‐tissue (+15%). In ‘CAC’ patients, a strong negative Spearman correlation (ρ = −0.8) was identified between <SUV_aorta_> and volumes of adipose‐tissue. The machine‐learning model identified ‘CAC’ at baseline with 81% of accuracy, highlighting <SUV_aorta_> of spleen, pancreas, liver, and adipose‐tissue as most relevant features. The model performance was suboptimal (54%) when classifying ‘Dev CAC’ versus ‘No CAC’.

**Conclusions:**

WB [^18^F]FDG‐PET/CT imaging reveals groupwise differences in the multi‐organ metabolism of LCP with and without CAC, thus highlighting systemic metabolic aberrations symptomatic of cachectic patients. Based on a retrospective cohort, our ML model identified patients with CAC with good accuracy. However, its performance in patients developing CAC was suboptimal. A prospective, multi‐centre study has been initiated to address the limitations of the present retrospective analysis.

## Introduction

Cancer‐associated cachexia (CAC) is a multifactorial syndrome characterized by a chronic disease‐related malnutrition with inflammation^1^ that disrupts metabolic homeostasis in cancer patients.^2^, ^3^ This debilitating condition is encountered in up to 50% of cancer patients.^2^ Among these, lung cancer (LC) stands as one of the most prevalent, where the incidence of cachexia is notably high (40–50%).^4^ CAC is clinically described as an ongoing, involuntary loss of skeletal muscle mass and functional impairment and is considered the primary cause of death in approximately 30% of cancer patients.^5^ Moreover, the presence of CAC is associated both with reduced tolerance to anticancer therapy and overall survival.^6^ The absence of a standardized, universally accepted definition presents challenges in generalizing findings across research populations.^7^

Since the introduction of the Fearon consensus in 2011,^6^ CAC is typically diagnosed when cancer patients lose more than 5% of their body weight within 6 months and exhibit a certain degree of systemic inflammation, by which time symptoms and cancer stage have already advanced to a critical stage. Because of this generic definition, treatment strategies should be multi‐professional and involve anti‐tumour treatment, nutritional interventions, psychological support, pharmacological interventions, physical exercise programs, and, in the end, best supportive care.^1^, ^2^, ^8^ The overarching goal is the reduction of systemic wasting by increasing both food intake and muscle mass to compensate for weight loss.^1^, ^2^, ^8^ However, management of CAC does not always provide sustained clinical benefits to patients, such as increased quality of life and cancer‐related survival. Although CAC can affect different systems including tissues, organs, and bones, the factors contributing to this condition remain ambiguous.^8^, ^9^ Therefore, novel approaches are needed to diagnose CAC in its early stages, before the onset of symptoms, at a stage called pre‐cachexia.

In order to detect CAC early, it is important to understand the underlying pathophysiology and its effects on the metabolism across multiple organs.^8^, ^9^ A simple, first approach, was the introduction of the body mass index (BMI)‐adjusted weight loss grading system (WLGS) that allowed to relate BMI and weight loss with the survival of cancer patients.^10^ Here, cancer patients undergo computed tomography (CT) imaging for adequate staging.^S1^ CT image‐based assessment of muscle and adipose tissue distribution can then help characterize body composition in cancer patients,^11^ ^,S2^ which has been shown to be beneficial in obese cancer patients for whom cachexia might easily be overlooked.^12^ More specifically to lung cancer patients (LCP), the recent TRACERx study demonstrated a relevant association between low CT‐derived areas (measured on the axial slice of the vertebra L3) of subcutaneous and visceral adipose tissue as well as skeletal muscle tissue, and reduced patient survival.^13^

In contrast to CT, whole‐body (WB) [^18^F]FDG‐PET is a unique imaging tool to observe and quantify metabolic processes across various organs throughout the human body, promising to detect systemic aberrations. Since its inception in the late 1990s,^14^ PET/CT has been accepted as a standard of care imaging modality in daily clinical routine, particularly in oncology for diagnosing cancer and monitoring disease progression. In the context of lung cancer, PET/CT has proven its potential in both diagnosis and treatment planning.^15^ However, its potential for detecting CAC remains underexplored with only a few studies available.^11^ For example, a decreased [^18^F]FDG uptake in the liver was found to be associated with anaemia, poor nutritional state and systemic inflammation, leading to a more probable CAC development and shorter overall survival.^16^ Elevated [^18^F]FDG‐derived metabolic tumour uptake was associated with a greater risk of malnutrition in LC patients, while no correlation was found between the metabolic tumour uptake and the CT‐measured body composition parameters.^17^ Further, subcutaneous adipose tissue volume negatively correlated with [^18^F]FDG uptake of LC, and, accordingly, higher volumes were associated with better progression‐free survival.^18^ However, these studies focused on specific regional uptake patterns, thereby overlooking the systemic nature of CAC. To address this shortfall, Jiang et al. recently investigated abnormal glucose metabolism across multiple tissues using [^18^F]FDG‐PET/CT in LCP with CAC.^19^

Given the systemic effects of CAC on whole‐body metabolism, FDG‐PET imaging is hypothesized to add value to the diagnostic work‐up of cancer patients at risk of developing CAC at an early stage so as to maximize the efficacy of therapeutic interventions directed not only toward the cancer but to the associated CAC as well. In this retrospective study with LCP, we analyse whole‐body FDG‐PET/CT imaging data and available lab parameters, both of which were acquired during the standard diagnostic workup of these patients, for additional insights into the status of CAC. We seek to detect distinctly different metabolic patterns and volume effects in whole‐body FDG‐PET/CT images of LCP without (‘No CAC’) and with CAC (‘CAC’), as well as in patients who will develop CAC (‘Dev CAC’) during follow‐up, and to generate machine‐learning based prediction models for each of these CAC categories. In this way, we hope to facilitate the early detection of CAC in cancer patients prior to evident signs or symptoms such as loss of appetite, muscle, or body weight, ultimately aiming to improve patient outcomes (quality of life and survival).

## Materials and methods

### Study subjects

This multi‐centre study comprised a retrospective cohort of LCP who had undergone WB [^18^F]FDG‐PET/CT examinations for their initial clinical staging. The acquisition of all data adhered strictly to the guidelines set forth in the Declaration of Helsinki, prioritizing ethical considerations and compliance with relevant legislation (Institutional Review Boards: 259/18‐ek, 21306_oss, 1649/2016). To safeguard patient confidentiality, all imaging data from the retrospective cohorts underwent complete anonymization.

In total, 490 WB PET/CT datasets (221 from the University Hospital of Leipzig, Germany, 199 from the Azienda Ospedaliero‐Universitaria Careggi in Florence, Italy, and 70 from the Medical University of Vienna, Austria) were included in this study. LCP were included if they were diagnosed with LC but treatment‐naive at the time of their baseline scan, and if their body weight and weight loss over the course of subsequent therapy were documented.

**Figure 1:**
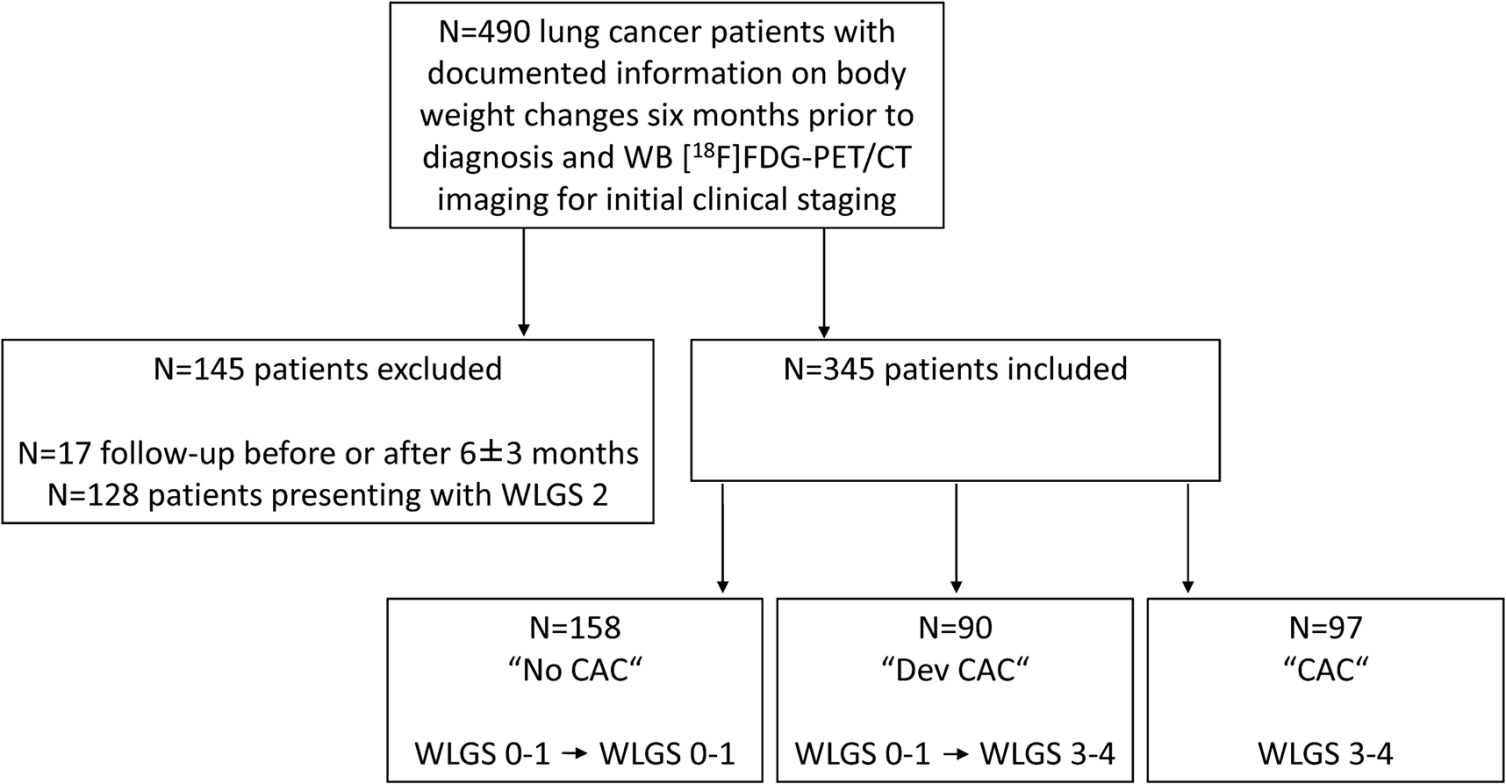
Flow chart for inclusion and stratification of lung cancer patients. [^18^F]FDG‐PET/CT, [^18^F]fluoro‐2‐deoxy‐D‐glucose positron emission tomography/computer tomography; CAC, cancer‐associated cacWB, whole‐body; WLGS, weight loss grading system.

Further, the cachectic status was assessed using the Weight Loss Grading System (WLGS), which classifies the body mass index (BMI) and the body weight loss during the past 6 months in five steps.^10^ ^,S3^ LCP exhibiting WLGS 0–1 at both baseline and first follow‐up were categorized as a non‐cachexia phenotype (‘No CAC’). LCP with WLGS 3–4 at baseline were classified as a CAC phenotype (‘CAC’). Patients displaying WLGS 0–1 at baseline and WLGS 3–4 at follow‐up were identified as being in the development of CAC (‘Dev CAC’). In total, 345 patients were classified in one of the three categories of CAC development and were considered for subsequent analysis (Figure 1). Individuals falling within the WLGS 2 were excluded to mitigate the inclusion of subjects whose weight changes may be attributed to factors other than CAC.

Demographic and clinical characteristics of patients were collected from the hospital medical records and examination report management systems, both at the baseline scan and during the first follow‐up (re‐staging), at 3‐ to 9‐month post‐baseline. Clinical LC stage was reported according to the 8th edition of the TNM classification for LC.^20^ Baseline blood values, including leukocytes (white blood cells count), serum creatinine, serum aspartate aminotransferase (ASAT), sodium, potassium, and total calcium, were available for *N* = 276 (80%) of the patients enrolled in the study. Furthermore, C‐reactive protein (CRP), triglycerides, cholesterol, proteins, and albumin were accessible from the clinical records of 100 (29%) patients enrolled. For these patients, the modified Glasgow prognostic score (mGPS) was assessed as a measure of the systemic inflammatory response:^21^ a score of 1 was assigned to patients with elevated CRP (>10 mg/L), and a score of 2 was given to patients with both elevated CRP and decreased serum albumin (<3.5 mg/L).

Survival data were retrieved and included the observation period, defined as the time from the date of patient enrolment (i.e., first diagnosis of LC with the date of PET/CT study) to the date of an event (i.e., death, lost‐to‐follow‐up), and the endpoint overall survival (OS). Survival analyses were conducted using the Kaplan–Meier estimator and log‐rank test to determine whether survival curves were significantly different. For the analysis, the software package GraphPad Prism (v10.2.1 for macOS, La Jolla, California, USA) was utilized.

### Imaging protocol

Image acquisitions were performed using four different PET/CT systems: Siemens Biograph 64 TruePoint (*N* = 70), Siemens mCT 16 (*N* = 221), Philips Gemini TF (*N* = 179), and GE HealthCare Discovery MI (*N* = 20). At all three sites, participants were asked to fast for 6 hours before the examinations. Each subject underwent a static PET acquisition in supine position following an intravenous injection of [^18^F]FDG (305 ± 66 MBq). Uptake times varied across the three sites, with an average of 75 ± 29 minutes post‐injection. PET images were reconstructed with attenuation and scatter corrections applied using the adjoined CT information.

### Image analysis

We report organ‐based image readouts in all patients. The CT was used to automatically segment multiple volumes of interest (VOIs) using the automated segmentation tool MOOSE.^22^ Each segmentation was manually verified by an experienced clinician and five medical students, by using the visualization software 3D Slicer.^23^ In particular, target regions known to be involved in maintaining systemic metabolic homeostasis were consequently considered for the subsequent analysis: spleen, kidneys, liver, pancreas, myocardium, skeletal muscle, subcutaneous, and visceral adipose tissue.^24^ ^,S4^ For muscle and adipose tissue segmentations, the axial region around the vertebra L3 was considered as reference.^25^ ^,S5^ Each VOI was subsequently overlapped on the corresponding PET image. Particular attention was paid that the organ segments were free from metastases. The mean standardized‐uptake‐values (SUVs) and the corresponding volumes were extracted (Figure S1). In order to standardize the quantitative PET information across the three different medical sites, SUVs were subsequently normalized to the aorta (including both blood pool and aortic wall) uptake (<SUV_aorta_>).

### Data analysis

Normality of continuous variables was assessed with a Shapiro–Wilk test. Imputing of null values was performed with a k‐nearest neighbour imputation.^26^ The Mann–Whitney U test and percentage differences were used for comparing the mean of blood parameters, age, BMI, SUV_aorta_, and volumes from each VOI between the phenotypical groups of ‘No CAC’, ‘Dev CAC’, and ‘CAC’. We used the chi‐square test for comparing categorical variables and considered *P*‐values ≤ 0.05 as statistically significant. Correlations within imaging parameters were studied with Spearman correlation analysis and visualized with chord plots.

We performed multivariate analyses to identify independent risk factors by training multiple machine‐learning models, including logistic regression, Extreme Gradient Boosting, CatBoost, and Extra Trees Classifiers. The model with the highest accuracy was then selected for binary classification between the two cohorts, ‘No CAC’ and ‘CAC’. This analysis was subsequently repeated for classification between ‘No CAC’ and ‘Dev CAC’, and between ‘No CAC’ and a merged ‘CAC Phenotype’ cohort (‘Dev CAC + CAC’). Ninety per cent of the data were allocated to the training set, while the remaining 10% were used as the test set for validation purposes. Only variables that were statistically different among the three cohorts were included in the training process and normalized to a range of 0 to 1. Imbalance in the data was addressed using the Synthetic Minority Oversampling Technique (SMOTE). The model's performance was evaluated using the area‐under‐the‐receiver‐operating‐characteristic (AUC) curve and by constructing a confusion matrix. We identified the key factors contributing to the personalized prediction of cachexia for each patient with the Explainable AI technique SHapley Additive exPlanations (SHAP) analysis.

## Results

### Demographics and clinical characteristics

Using the WLGS, 158/345 (46%) patients were classified as ‘No CAC’, 90/345 (26%) as ‘Dev CAC’, and 97/345 (28%) as ‘CAC’. Details of the patient demographics and tumour characteristics are summarized in Table 1. The demographic characteristics of the LC cohort, including age at diagnosis, as well as the distribution of sex, tumour stage, and histology types, align with the distributions typically observed in clinical practice.^27^, ^28^ Patients in the ‘CAC’ group had a median BMI of 23 kg/m^2^, which was significantly lower (*P* < 0.001) than in the ‘No CAC’ (26 kg/m^2^) and the ‘Dev CAC’ cohort (24 kg/m^2^). The prevalence of metastasized LC (stage IV) was highest in the ‘Dev CAC’ (49%) and the lowest in the ‘No CAC’ (26%). Out of the entire cohort (*N* = 345), information on cancer stages was not available for 63 (18%) patients. Similarly, information on 1‐year overall survival was missing for 43% of the patients.

**Table 1:** Demographics of lung cancer patients according to their cachexia status

Parameter	Total	No CAC	Dev CAC	CAC	*P*‐value	*P*‐value
	*N* = 345	*N* = 158	*N* = 90	*N* = 97	No CAC vs. Dev CAC	No CAC vs. CAC
	100%	46%	26%	28%		
Age, years, (range)	69 (33–89)	70 (33–88)	70 (34–85)	67 (38–89)	0.56	0.01*
Sex, F/M	116 (34%) /229 (66%)	51 (32%) /107 (68%)	34 (38%) /56 (62%)	31 (32%) /66 (68%)	0.38	0.95
BMI, kg/m^2^ (range)	25 (14–58)	26 (19–43)	24 (15–58)	23 (14–31)	<0.01**	<0.01**
≤18.5	17 (5%)	0 (0%)	7 (8%)	10 (10%)		
18.6–24.9	167 (48%)	60 (38%)	49 (54%)	58 (60%)		
25.0–29.9	108 (31%)	59 (37%)	25 (28%)	24 (25%)		
≥30	53 (16%)	39 (25%)	9 (10%)	5 (5%)		
Tumour stage					<0.01**	<0.01**
I	71 (21%)	43 (28%)	13 (14%)	15 (16%)		
II	38 (11%)	23 (14%)	6 (7%)	9 (9%)		
III	51 (15%)	27 (17%)	18 (20%)	6 (6%)		
IV	122 (35%)	42 (26%)	44 (49%)	36 (37%)		
Missing	63 (18%)	23 (15%)	9 (10%)	31 (32%)		
Histology					0.20	<0.01**
ADC	169 (49%)	88 (56%)	42 (47%)	39 (40%)		
LCC	11 (3%)	2 (1%)	4 (4%)	5 (5%)		
NSCLC‐NOS	22 (6%)	10 (6%)	9 (10%)	3 (3%)		
SCC	92 (27%)	44 (28%)	24 (27%)	24 (25%)		
SCLC	16 (5%)	5 (3%)	6 (7%)	5 (5%)		
Missing	35 (10%)	9 (6%)	5 (5%)	21 (22%)		

Data are shown as number of patients with percentage (%), unless otherwise specified. Significant differences are highlighted with asterisks.

ADC, adenocarcinoma; BMI, body mass index; CAC, cancer‐associated cachexia; Dev, developing; F, female, LCC, large cell carcinoma; M, male; N, number of patients; NSCLC‐NOS ‐ non‐small cell lung cancer ‐ not otherwise specified; SCC, squamous cell carcinoma; SCLC, small cell lung cancer.

* *P* < 0.05.

** *P* < 0.01.

The clinical characteristics of the LCP are summarized in Table 2. No significant differences were observed in the blood tests among the three cohorts, except for leukocytes, serum creatinine levels, and serum ASAT levels. Specifically, leukocytes and creatinine were significantly lower in the ‘CAC’ group (*P* < 0.01), while ASAT levels were slightly higher in the ‘CAC’ group. In addition, cholesterol levels were lower (*P* = 0.05) in the ‘CAC’ group compared with the ‘No CAC’.

**Table 2:** Summary of blood values of lung cancer patients according to their cachexia status

Total dataset (*N* = 276)	No CAC (*N* = 122)	Dev CAC (*N* = 78)	CAC (*N* = 76)	*P*‐value	*P*‐value
				No CAC vs. Dev CAC	No CAC vs. CAC
Leukocytes (cells/nL)	7.6 (4.0–15.7)	7.7 (3.4–15.4)	6.8 (2.9–14.8)	0.96	0.01*
Creatinine (μmol/L)	69.9 (25.0–129.0)	65.2 (25.0–121.0)	60.0 (25.0–105.0)	0.09	<0.01**
ASAT (μkat/L)	0.3 (0.1–0.6)	0.3 (0.1–0.6)	0.4 (0.2–0.6)	0.27	<0.01**
Sodium (mmol/L)	141.0 (134.0–147.0)	141.4 (134.0–146.0)	141.0 (137.0–145.8)	0.80	0.58
Potassium (mmol/L)	4.3 (3.0–5.5)	4.4 (2.9–6.0)	4.5 (3.0–6.0)	0.09	0.19
Total calcium (mmol/L)	2.3 (2.0–2.6)	2.3 (2.1–2.6)	2.4 (2.1–2.8)	0.60	0.03

Available dataset (*N* = 100)	No CAC (*N* = 30)	Dev CAC (*N* = 18)	CAC (*N* = 52)	*P*‐value	*P*‐value
				No CAC vs. Dev CAC	No CAC vs. CAC
CRP (mg/L)	12.0 (0.5–308.0)	6.2 (0.5–134.0)	15.5 (0.5–220.8)	0.42	0.96
Triglycerides (mol/L)	0.9 (0.6–6.7)	1.0 (0.5–7.4)	1.0 (0.4–5.3)	0.69	0.33
Cholesterol (mmol/L)	5.2 (1.0–7.6)	5.0 (1.3–7.6)	4.8 (2.1–7.6)	0.54	0.05*
Protein (g/L)	60.8 (31.0–80.6)	60.8 (41.4–70.2)	61.1 (44.8–80.6)	0.70	0.40
Albumin (g/L)	38.1 (0.4–47.3)	38.1 (0.7–44.8)	38.1 (26.3–45.0)	0.98	0.91
mGPS, *N* (%)				0.97	0.28
0	22 (73%)	12 (67%)	29 (56%)		
1	5 (17%)	4 (22%)	14 (27%)		
2	3 (10%)	2 (11%)	9 (17%)		

Results are reported as median and minimum‐maximum range. Significant differences are highlighted with asterisks.

ASAT, aspartate aminotransferase; CAC, cancer‐associated cachexia; CRP, C‐reactive protein; Dev, developing; mGPS, modified Glasgow prognostic score.

* *P* < 0.05.

** *P* < 0.01.

The mGPS differed between the three groups according to the metabolic condition. In the ‘No CAC’ group, 73% of LCP had an mGPS = 0 compared with 67% and 56% in ‘Dev CAC’ and ‘CAC’ groups, respectively. In contrast, 10% of LCP in ‘No CAC’ had an mGPS = 2 compared with 11% and 17% in the ‘Dev CAC’ and in the ‘CAC’ group, respectively.

Median overall survival of LCP differed significantly between the groups of ‘No CAC’, ‘Dev CAC’, and ‘CAC’ with 684, 311, and 513 days, respectively (*P* < 0.001). LCP who developed cachexia following the LC diagnosis had the lowest survival of the three groups (Figure S2).

### Imaging readouts

**Figure 2:**
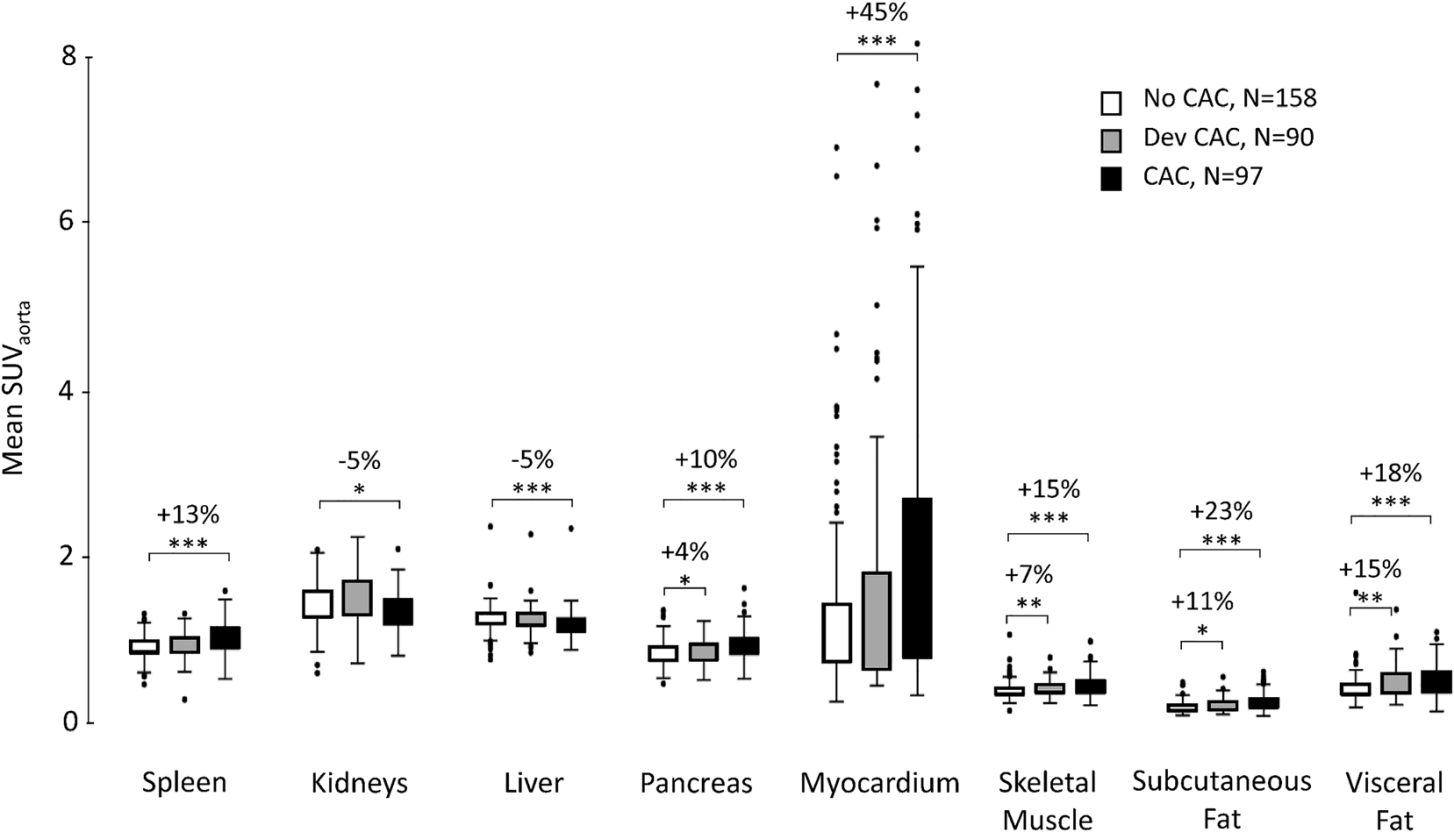
Mean SUV_aorta_ distributions in target organs for ‘No CAC’ (white), ‘Dev CAC’ (grey) and ‘CAC’ (black) cohorts. Significant differences are indicated with stars (*P < 0.05, **P < 0.01, ***P < 0.001).

<SUV_aorta_> varied between a minimum of 0.2 ± 0.1 (subcutaneous adipose tissue) and a maximum 2.2 ± 2.1 (myocardium) across the three cohorts and target organs (Figure 2). The ‘CAC’ cohort exhibited significantly higher <SUV_aorta_> (*P* < 0.01) in all target regions, except the kidneys and the liver, where the <SUV_aorta_> was lower compared with the ‘No CAC’ group. The ‘Dev CAC’ cohort exhibited a small but significant increase in <SUV_aorta_> in the pancreas (4%), skeletal muscle (7%), and adipose tissue regions (11%) compared with the ‘No CAC’ group (Table 3).

**Table 3:** Mean SUV_aorta_ and volumes in key organs of lung cancer patients according to their cachexia status

	No CAC (*N* = 158)	Dev CAC (*N* = 90)	CAC (*N* = 97)	%‐difference	%‐difference
				No CAC vs. Dev CAC	No CAC vs. CAC
Mean SUV_aorta_					
Spleen	0.93 ± 0.14	0.96 ± 0.14	1.06 ± 0.19	3%	13%
Kidneys	1.44 ± 0.25	1.55 ± 0.33	1.36 ± 0.23	7%	−5%
Liver	1.27 ± 0.15	1.26 ± 0.16	1.21 ± 0.17	−1%	−5%
Pancreas	0.86 ± 0.16	0.89 ± 0.15	0.95 ± 0.19	4%	10%
Myocardium	1.38 ± 1.18	1.57 ± 1.48	2.18 ± 2.12	13%	45%
Skeletal muscle	0.41 ± 0.09	0.43 ± 0.10	0.48 ± 0.15	7%	15%
Subcutaneous fat	0.21 ± 0.07	0.23 ± 0.08	0.26 ± 0.11	11%	23%
Visceral fat	0.44 ± 0.15	0.51 ± 0.19	0.53 ± 0.19	15%	18%
Volumes (cm^3^)					
Spleen	225 ± 127	193 ± 96	223 ± 155	−16%	−2%
Kidneys	317 ± 92	293 ± 99	321 ± 80	−8%	1%
Liver	1538 ± 450	1445 ± 461	1562 ± 359	−6%	2%
Pancreas	73 ± 23	62 ± 24	66 ± 23	−16%	−11%
Myocardium	131 ± 34	116 ± 34	123 ± 33	−12%	−6%
Skeletal muscle	817 ± 234	714 ± 199	726 ± 219	−13%	−12%
Subcutaneous fat	1145 ± 550	947 ± 535	753 ± 471	−19%	−41%
Visceral fat	1012 ± 565	806 ± 531	661 ± 463	−23%	−42%

Data are shown as mean ± standard deviation.

CAC, cancer‐associated cachexia; Dev, developing; SUV, standardized uptake value.

Mean volumes of the target organs ranged from (62 ± 24) cm^3^ in the pancreas to (1562 ± 359) cm^3^ in the liver (Table 3). Patients in the ‘CAC’ group had smaller organ volumes (*P* < 0.01) of the pancreas (11%), skeletal muscle (12%), subcutaneous adipose tissue (41%), and visceral adipose tissue (42%), compared with the ‘No CAC’ group. The ‘Dev CAC’ group also exhibited smaller volumes as compared with the ‘No CAC’ group, in all regions except the spleen and visceral adipose tissue, with the greatest percentage in difference of 23% observed in the visceral adipose tissue (Figure 2, Table 3).

**Figure 3:**
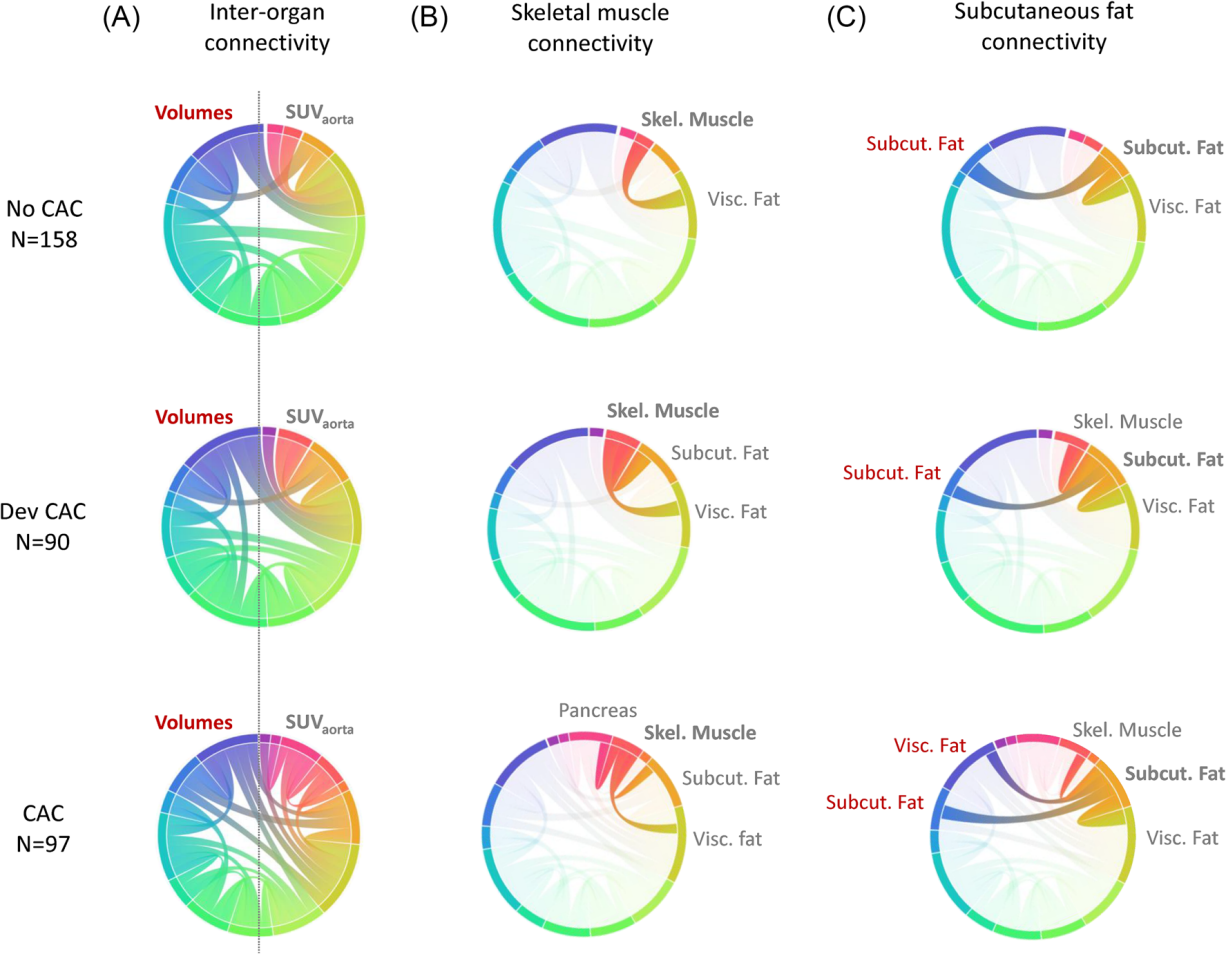
Correlations among imaging parameters (volumes and <SUV_aorta_>) of the target regions in the ‘No CAC’ (upper), ‘Dev CAC’ (middle) and ‘CAC’ (lower) cohorts. In the chord plots, external nodes represent the imaging parameters, while the thickness of the internal curves indicates the strength of the correlation. (A) All significant correlations between the parameters and regions considered. (B, C) Connectivity profiles of subcutaneous adipose tissue <SUV_aorta_> and visceral adipose tissue <SUV_aorta_>, respectively. SUV, standardized uptake value.

The significant Spearman correlations (ρ ≥ 0.5) are depicted for the three cohorts in Figure 3. The number of significant correlations increased from the ‘No CAC’ to the ‘CAC’ group (Figure 3A). A similar trend was observed in the connectivity profiles (<SUV_aorta_>) of skeletal muscle (Figure 3B) and subcutaneous adipose tissue (Figure 3C). Specifically, in the ‘No CAC’ cohort, Spearman's correlation indicated a moderate positive correlation (ρ = 0.5) between the <SUV_aorta_> of skeletal muscle and subcutaneous adipose tissue. In both the ‘Dev CAC’ and ‘CAC’ cohorts, a moderate positive correlation existed between the <SUV_aorta_> of skeletal muscle and subcutaneous adipose tissue, as well as between subcutaneous and visceral adipose tissue (ρ = 0.6, respectively). In the ‘CAC’ group, a moderate positive correlation was seen between <SUV_aorta_> of skeletal muscle and pancreas (ρ = 0.6), as well as between visceral adipose tissue and pancreas (ρ = 0.6). Finally, a moderate and a strong negative correlation between <SUV_aorta_> and volumes of subcutaneous and visceral adipose tissue regions were found in the ‘Dev CAC’ cohort (ρ = −0.6) and in the ‘CAC’ cohort (ρ = −0.8), respectively.

### Multivariate regression analysis

**Figure 4:**
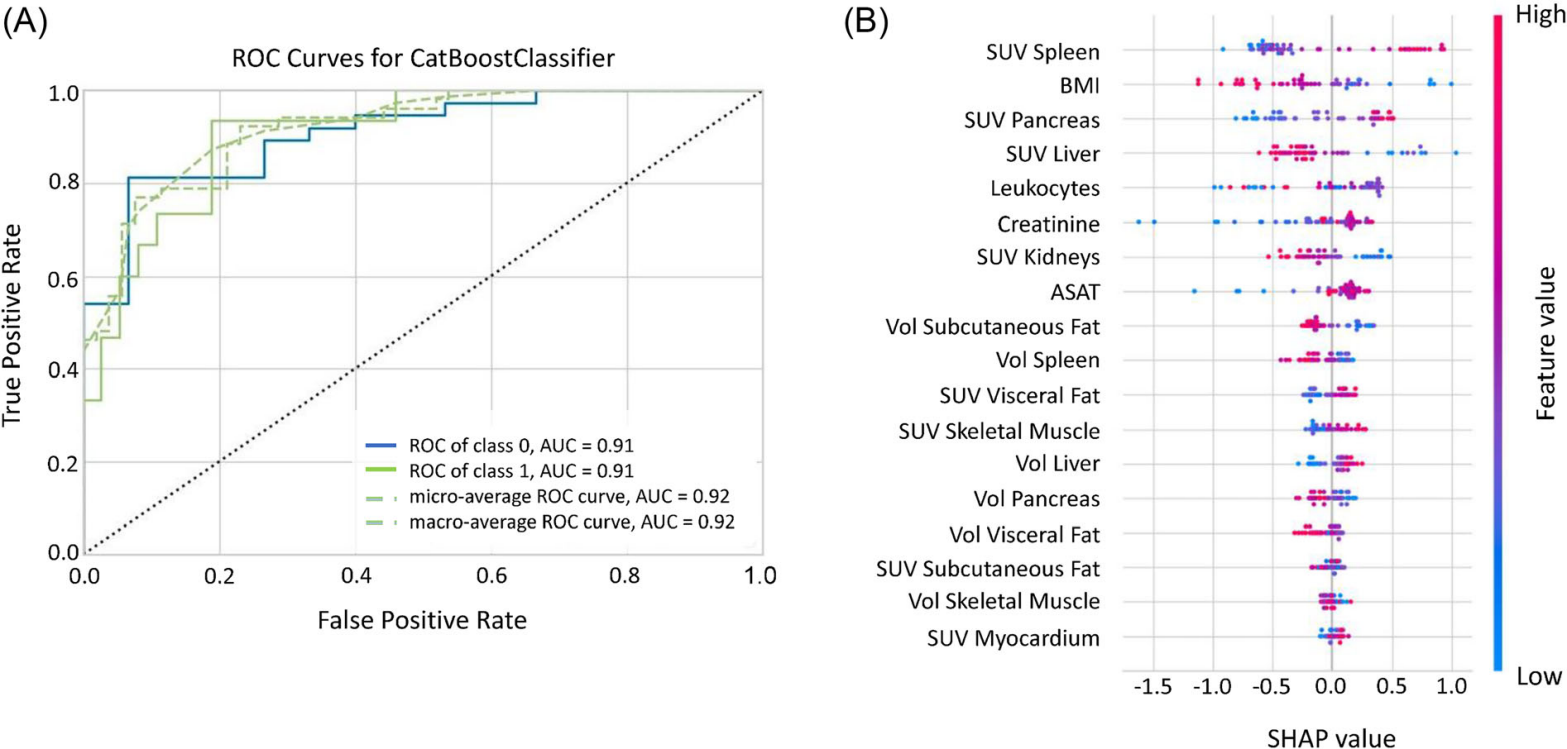
CatBoost classifier ROC curve (A) and SHAP analysis (B) for the binary classification between ‘No CAC’ and ‘CAC’ cohorts. The position of the dots to the left or right in the SHAP plot (B) indicates their influence toward a ‘No CAC’ or ‘CAC’ classification, respectively. The colour of the dots indicates the absolute value of each feature: Blue for lower and pink for higher values. ASAT, aspartate aminotransferase; BMI, body mass index, SUV, standardized uptake value; Vol, volume.

The machine‐learning models for binary classification between ‘CAC’ and ‘No CAC’ included the following variables: imaging parameters (<SUV_aorta_> and volumes), BMI, leukocytes, creatinine, and ASAT. Among the trained models, the CatBoost Classifier yielded the highest values for accuracy (81%), precision (82%), and AUC (0.91) (Figure 4A). Overall, 81% of LCP without cachexia were correctly classified as ‘No CAC’, while 73% were correctly classified as ‘CAC’ by the machine‐learning model.

The SHAP analysis identified the independent features critical for prediction of CAC (Figure 4B). Here, low BMI, high <SUV_aorta_> of spleen and pancreas, and low <SUV_aorta_> of the liver were the most relevant characteristics of cachexia. Other features, such as the <SUV_aorta_> in the kidney, the visceral adipose tissue, and the skeletal muscle, as well as the subcutaneous adipose tissue volume, displayed distinct absolute values but exhibited less overall impact on the model. Low <SUV_aorta_> of kidneys, small adipose tissue volume, and high <SUV_aorta_> of visceral adipose tissue and skeletal muscle were indicative of CAC.

**Figure 5:**
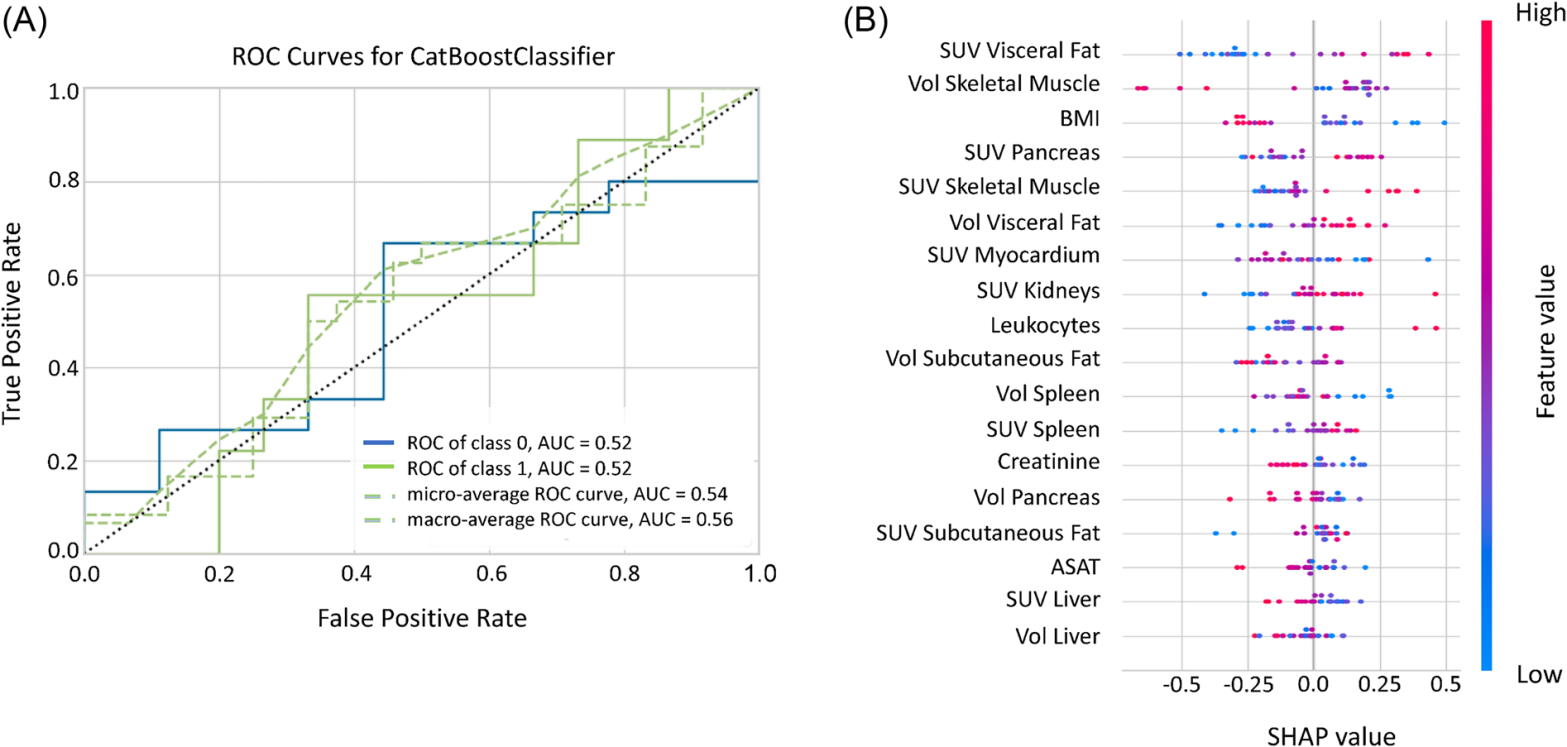
CatBoost classifier ROC curve (A) and SHAP analysis (B) for the binary classification between ‘No CAC’ and ‘Dev CAC’ cohorts. The position of the dots to the left or right in the SHAP plot (B) indicates their influence toward a ‘No CAC’ or ‘Dev CAC’ classification, respectively. The colour of the dots indicates the absolute value of each feature: Blue for lower and pink for higher values. ASAT, aspartate aminotransferase; BMI, body mass index, SUV, standardized uptake value; Vol, volume.

The binary classification, repeated with the ‘Dev CAC’ cohort against ‘No CAC’, did result in a lower accuracy (54%), precision (50%), and AUC (0.52) (Figure 5A). The SHAP analysis again identified high <SUV_aorta_> of visceral adipose tissue, skeletal muscle and pancreas, low BMI, as well as small volumes of the skeletal muscle, and subcutaneous adipose tissue as independent predictors of CAC (Figure 5B). An additional classification of ‘No CAC’ versus ‘CAC Phenotype’ (i.e., ‘Dev CAC’ and ‘CAC’) resulted again in a reduced performance compared with the binary classification between ‘CAC’ and ‘No CAC’ (58% accuracy, 63% precision, and 0.65 AUC; Figure S4).

## Discussion

CAC is a complex syndrome with delayed diagnosis, at a stage when physical weight loss is already irreversible. Therefore, novel approaches to detecting CAC at its onset are needed to help facilitate improved patient outcomes.^3^, ^7^ The goal of this retrospective, multi‐centre study across three European clinical sites was a comprehensive analysis of WB [^18^F]FDG‐PET/CT image data of LCP to identify metabolic patterns associated with CAC, in particular at early stage. Our analysis demonstrated distinct metabolic patterns in the three groups representing CAC development, with increased metabolic activity (<SUV_aorta_>) in most of the target organs among patients presenting with CAC at baseline (‘CAC’; Figure 2, Table 3). Reduced <SUV_aorta_> were observed in the liver and kidneys (Figure 2, Table 3). Furthermore, we found a negative correlation between <SUV_aorta_> and volumes of adipose tissue regions in LCP with CAC at baseline. The presence of CAC did critically affect survival of LCP; LCP who had (‘CAC’) or developed CAC (‘Dev CAC’) at the time of LC diagnosis had the poorest median overall survival compared with those LC who did not develop CAC (Figure S2).

Patients developing CAC exhibited higher <SUV_aorta_> in the pancreas, muscles, and adipose tissue compared with patients without CAC. An increase in the number of significant correlations between <SUV_aorta_> and volumes of the target organs also appeared in the ‘Dev CAC’ group compared with ‘No CAC’ (Figure 3), specifically between subcutaneous adipose tissue and both visceral adipose tissue and skeletal muscle mass (Figure 3C). Our explainable machine‐learning model yielded an accuracy of 81% in detecting CAC (Figure 4). This accuracy was markedly reduced (54%) for classifying ‘Dev CAC’ against ‘No CAC’ (Figure 5). We assume that the heterogeneity of our retrospective clinical training dataset did contribute to this performance, which limits the applicability of this particular model in detecting CAC in an individual lung cancer patient during FDG‐PET‐based staging.

Increased splenic [^18^F]FDG uptake, as seen especially in the ‘CAC’ group (Figure 2, Table 3), is associated with inflammation, but the spleen also plays a crucial role in metabolic control, including lipid metabolism.^29^ Likewise, the pancreas is a vital metabolic organ responsible for maintaining glucose homeostasis through the secretion of insulin, glucagon and a variety of digestive enzymes; heightened SUV levels in the pancreas are linked to pancreatitis.^30^ The liver is crucial for regulating energy metabolism and nutrient storage, but in contrast to the other two metabolic organs we observed a slight reduction in liver uptake in the ‘CAC’ group, potentially influenced by altered glucose uptake in hepatic cells in the presence of metastatic cancer (49% in the ‘Dev CAC’ and 37% in ‘CAC’, Table 1).

In particular, the Cori cycle might be an important mechanism that can explain the decreased hepatic glucose uptake in LCP with CAC due to increased hepatic gluconeogenesis stemming from lactate.^31^ In these LCP, lactate can originate from the tumour or the skeletal muscle cells. The Cori cycle as a shuttle system of lactate and glucose is energetically inefficient due to the cost of hepatic gluconeogenesis (6 adenosine triphosphate [ATP]) relative to energy yield by production of lactate from glycolysis in the muscle tissue (2 ATP). Nevertheless, it allows muscle activity to be maintained in conditions of extreme energetic stress.^32^ There is evidence of altered glycolytic and lactate metabolites, enzyme activity and transporter protein expression in liver, muscle, and tumour tissue during CAC.^32^ Changes in these three energy‐regulating tissues suggest ongoing substrate shuttling that may contribute to tumour growth, energetic inefficiency and therefore, unintended weight loss in patients with CAC. However, these explanations remain to be proven in further studies.

Patients with CAC exhibited significantly higher uptake in subcutaneous adipose tissue (23%), visceral adipose tissue (18%), and skeletal muscle (15%) compared with the ‘No CAC’ cohort (Table 3). Glucose metabolism for lipogenesis activated by CAC can contribute to increased FDG uptake in adipose tissue, and increased glucose uptake in adipose tissue regions may signify inflammation.^19^ ^,S6^ Further, heightened uptake in muscle mass could indicate proteolysis triggered by CAC, leading to muscle degradation, loss of muscle quantity and function, muscle strength, and thus sarcopenia.^33^

The observed systemic inflammation pattern in ‘CAC’ was supported by the available blood values, particularly the differences observed in CRP serum concentrations and the mGPS (Table 2). LCP with CAC at baseline exhibited elevated CRP levels, although not significantly different from the ‘No CAC’ group, and the percentage of patients with mGPS = 2 was higher with the development of CAC and in the ‘CAC’ cohort (17%, Table 2). The ‘No CAC’ group had instead the highest percentage of patients with mGPS = 0 (73%), indicating a lower systemic inflammatory response. Low creatinine levels are also associated with systemic inflammation and muscles degradation, a hallmark of CAC progression.^34^ ^,S7^ The survival analysis revealed that LCP in the ‘Dev CAC’ cohort had the lowest overall survival of the three groups (Figure S2). This group also had the highest fraction of patients with stage IV tumours (49%, Table 1), a confounder that may contribute to the worse prognosis of these patients compared with the ‘CAC ‘cohort.

Our study identified group‐wise metabolic patterns associated with the different stages of CAC, consistent with its systemic nature as a metabolic syndrome affecting multiple organs (Figure 3). While we were able to identify organs most affected by the disease, the significant variability in organ‐based <SUV_aorta_> did not permit an individualized detection of early CAC (‘Dev CAC’). Indeed, the observed increases in glucose uptake across most regions of this retrospective cohort were not clinically indicative in the ‘CAC’ groups (Figure 2), as the SUVs fell within the normal ranges of uptakes reported.^35^ This was also exemplified by the machine learning model, which detected cachexia (‘CAC’) with high accuracy (81%, Figure 4) but failed to reliably detect early stages of CAC (‘Dev CAC’) with an accuracy of only 54% (Figure 5). Due to the relatively small number of patients developing CAC used for training the machine learning model, we were unable to identify common patterns in clinical or imaging parameters of the patients correctly identified as being in the process of developing CAC (50% of the test set).

Therefore, and given the similarity in imaging parameters between the ‘Dev CAC’ and ‘No CAC’ groups, we attempted to improve predictive performance by grouping all patients with a CAC phenotype (‘Dev CAC’ + ‘CAC’). The comparison of <SUV_aorta_> distributions in key organs between the ‘No CAC’ and ‘CAC Phenotype’ groups led to better differentiation than the comparison between the ‘No CAC’ and ‘Dev CAC’ groups alone (Figure S3). The accuracy of the machine‐learning model also slightly improved to 58% (Figure S4), with 63% of LCP without CAC being correctly identified and 53% accurately classified as having the ‘CAC Phenotype’.

To reduce the complexity of our machine learning model and avoid possible overfitting, which may lead to lower performance, we repeated our binary classification of ‘No CAC’ versus ‘CAC’ by only considering BMI, mean SUV_aorta_ and volumes and excluding the blood parameters (Figure S5). The best‐performing model, an Extreme Gradient Boosting Classifier, achieved an accuracy of 73%, a precision of 81%, and an AUC of 0.75. This confirms that the combination of both multiple imaging and non‐imaging parameters remains a better tool for cachexia detection (81%, 82%, and 0.91).

Although we could observe different metabolic patterns in the FDG‐PET/CT images of the three groups of LCP, our model performance was limited. The challenge of identifying early stages of CAC may be exacerbated by the several limitations inherent to this study.^36^ Our data originated from three different medical centres, and exhibited a high heterogeneity, both in acquisition protocols and uptake times, as well as in the PET/CT systems employed on site. Moreover, unexpected variability in the dietary status could affect glycolysis in specific organs, such as the myocardium, and impair the reliability of mean SUV quantification. This could require a preliminary assessment of the uptake pattern, for instance according to what is already required in the setting of the diagnosis of cardiac sarcoidosis.^37^ Repeating the analysis with homogeneous data from a prospective setting, including defined imaging protocols, comprehensive blood records, and detailed information on weight loss, nutritional status, and survival, may enhance the robustness of our results.

Among the *N* = 345 patients with documented weight loss information, misclassification of CAC status both at baseline and at the first follow‐up could not be excluded. Weight loss was documented based on patients' self‐reports, and a standardized method for measuring weight was lacking. Previous studies assessed the reliability of self‐reported weight loss in clinical scenarios, finding significant differences between self‐reported and measured data, even though clinically irrelevant.^38^ In many cases, other information on malnutrition status, such as the Malnutrition Screening Tool, the Nutritional Risk Screening 2002, or the Malnutrition Universal Screening Tool was unavailable, despite evidence suggesting their potential utility in the diagnosis of CAC.^39^ ^,S8^

Finally, in the present study, SUV quantification was conducted as an average across the entire organ region, potentially ignoring metabolic abnormalities occurring at a smaller scale. The identification of potential metabolic aberrations from normal metabolic activity patterns at a voxel level using [^18^F]FDG‐PET/CT imaging may offer more detailed insights into altered metabolism in patients.^40^

## Conclusion

Our analysis of WB [^18^F]FDG‐PET/CT images of LCP revealed significant group differences in metabolic uptake patterns across relevant target organs. Higher <SUV_aorta_> of the spleen, pancreas, skeletal muscle, and visceral adipose tissue, and lower <SUV_aorta_> of the liver were found to be the most relevant indicators of CAC in LCP. Purpose‐built explainable machine learning‐based predictions demonstrated high accuracy in distinguishing between patients with (‘CAC’) and without CAC (‘No CAC’). However, the identification of CAC at early stages (‘Dev CAC’) in individual patients of our cohort was not possible with sufficient accuracy, most likely due to the substantial protocol‐based variability in this retrospective cohort, which is currently being addressed in a follow‐up prospective study employing multi‐centric, protocol harmonization.

## Conflict of interest

Daria Ferrara, Elisabetta M. Abenavoli, Thomas Beyer, Stefan Gruenert, Marcus Hacker, Swen Hesse, Lukas Hofmann, Smilla Pusitz, Michael Rullmann, Osama Sabri, Peter Sandøe, Roberto Sciagrà, Lalith Kumar Shiyam Sundar, Anke Tönjes, Hubert Wirtz, Josef Yu, and Armin Frille declare that they have no conflict of interest.

## Supporting information


**Figure S1.** Example of liver delineation in (a) coronal and (b) axial views in a CT image (left column). On the right column, the segmentation is fused with the corresponding PET image, and quantitative parameters (mean SUV_aorta_) are extracted.
**Figure S2.** Kaplan–Meier curves of survival times from lung cancer patients according to their cachexia status. A log‐rank test was performed, and a hazard ratio was calculated according to Mantel–Haenszel. Of note, in panel a), the lower survival rates in the ‘Dev CAC’ cohort can be explained by the higher frequency of advanced tumour stages in this category of patients. Panel b) shows the overall survival of lung cancer patients who have been diagnosed with cachexia during their trajectory of the disease. CAC = cancer associated cachexia, Dev = developing; mOS = median overall survival
**Figure S3.** Mean SUV_aorta_ distributions in target organs for ‘No CAC’ (white) and the grouped ‘Dev CAC + CAC’ (black) cohorts. Significant differences are indicated with stars (*P < 0.05, **P < 0.01, ***P < 0.001)
**Figure S4.** CatBoost Classifier ROC curve (a) and SHAP analysis (b) for the binary classification between ‘No CAC’ and ‘CAC Phenotype’ (‘Dev CAC’ + ‘CAC’) cohorts. The position of the dots to the left or right in the SHAP plot (b) indicates their influence toward a ‘No CAC’ or ‘CAC Phenotype’ classification, respectively. The colour of the dots indicates the absolute value of each feature: blue for lower and pink for higher values. ASAT = aspartate aminotransferase; BMI = body mass index, SUV = standardized uptake value; Vol = volume
**Figure S5.** XGBoost Classifier ROC curve (a) and SHAP analysis (b) for the binary classification between ‘No CAC’ and ‘CAC’ cohorts, including only BMI and imaging parameters. The position of the dots to the left or right in the SHAP plot (b) indicates their influence toward a ‘No CAC’ or ‘CAC’ classification, respectively. The colour of the dots indicates the absolute value of each feature: blue for lower and pink for higher values. BMI = body mass index, SUV = standardized uptake value; Vol = volume

**Data S1.** Supporting Information
