# Supplementary material for: Detection of cancer‐associated cachexia in lung cancer patients using whole‐body [18F]FDG‐PET/CT imaging: A multi‐centre study
Source: J Cachexia Sarcopenia Muscle. 2024 Aug 27;15(6):2375–86. doi: 10.1002/jcsm.13571 (PMC11634466; doi:10.1002/jcsm.13571)
Supplement: Supplementary file 1 — Figure S1. Example of liver delineation in (a) coronal and (b) axial views in a CT image (left column). On the right column, the segmentation is fused with the corresponding PET image, and quantitative parameters (mean SUVaorta) are extracted. Figure S2. Kaplan–Meier curves of survival times from lung cancer patients according to their cachexia status. A log‐rank test was performed, and a hazard ratio was calculated according to Mantel–Haenszel. Of note, in panel a), the lower survival rates in the ‘Dev CAC’ cohort can be explained by the higher frequency of advanced tumour stages in this category of patients. Panel b) shows the overall survival of lung cancer patients who have been diagnosed with cachexia during their trajectory of the disease. CAC = cancer associated cachexia, Dev = developing; mOS = median overall survival Figure S3. Mean SUVaorta distributions in target organs for ‘No CAC’ (white) and the grouped ‘Dev CAC + CAC’ (black) cohorts. Significant differences are indicated with stars (*P < 0.05, **P < 0.01, ***P < 0.001) Figure S4. CatBoost Classifier ROC curve (a) and SHAP analysis (b) for the binary classification between ‘No CAC’ and ‘CAC Phenotype’ (‘Dev CAC’ + ‘CAC’) cohorts. The position of the dots to the left or right in the SHAP plot (b) indicates their influence toward a ‘No CAC’ or ‘CAC Phenotype’ classification, respectively. The colour of the dots indicates the absolute value of each feature: blue for lower and pink for higher values. ASAT = aspartate aminotransferase; BMI = body mass index, SUV = standardized uptake value; Vol = volume Figure S5. XGBoost Classifier ROC curve (a) and SHAP analysis (b) for the binary classification between ‘No CAC’ and ‘CAC’ cohorts, including only BMI and imaging parameters. The position of the dots to the left or right in the SHAP plot (b) indicates their influence toward a ‘No CAC’ or ‘CAC’ classification, respectively. The colour of the dots indicates the absolute value of each feature: blue for lower a [file JCSM-15-2375-s001.docx]

**SUPPLEMENTARY MATERIALS**


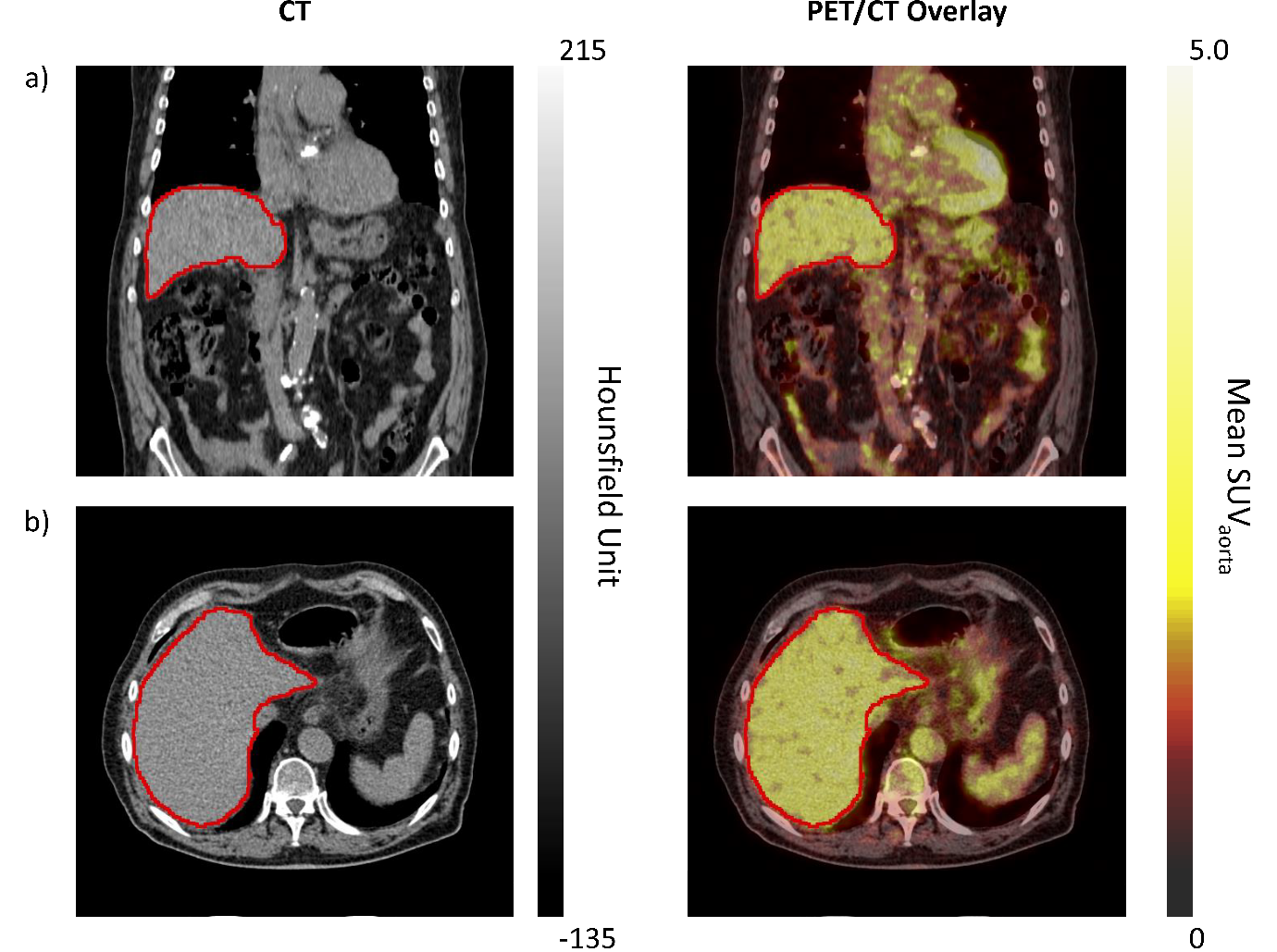


**Fig. S1** Example of liver delineation in (a) coronal and (b) axial views in a CT image (left column). On the right column, the segmentation is fused with the corresponding PET image, and quantitative parameters (mean SUV_aorta_) are extracted.


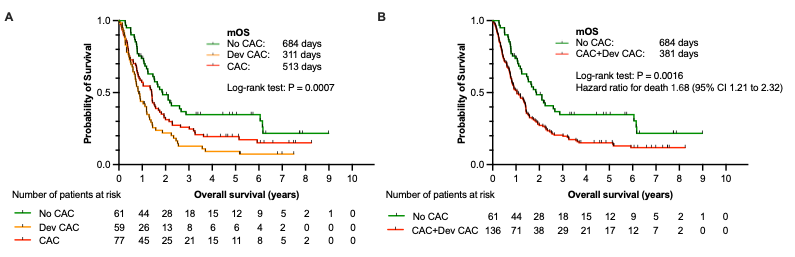


b)

a)

**Fig. S2** Kaplan-Meier curves of survival times from lung cancer patients according to their cachexia status. A log-rank test was performed, and a hazard ratio was calculated according to Mantel-Haenszel. Of note, in panel a), the lower survival rates in the “Dev CAC” cohort can be explained by the higher frequency of advanced tumor stages in this category of patients. Panel b) shows the overall survival of lung cancer patients who have been diagnosed with cachexia during their trajectory of the disease. CAC = cancer associated cachexia, Dev = developing; mOS = median overall survival.


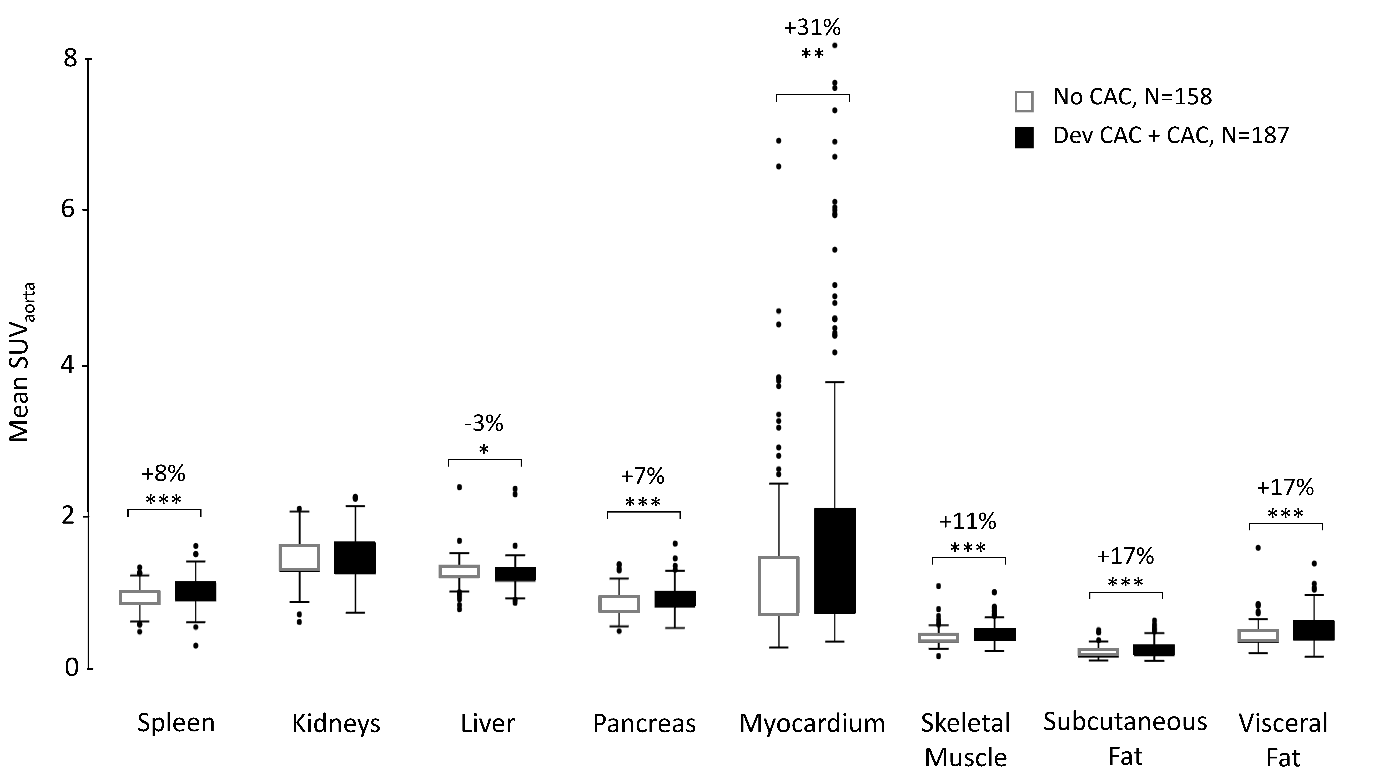


**Fig. S3** Mean SUV_aorta_ distributions in target organs for “No CAC” (white) and the grouped “Dev CAC + CAC” (black) cohorts. Significant differences are indicated with stars (*P<0.05, **P<0.01, ***P<0.001)**.**


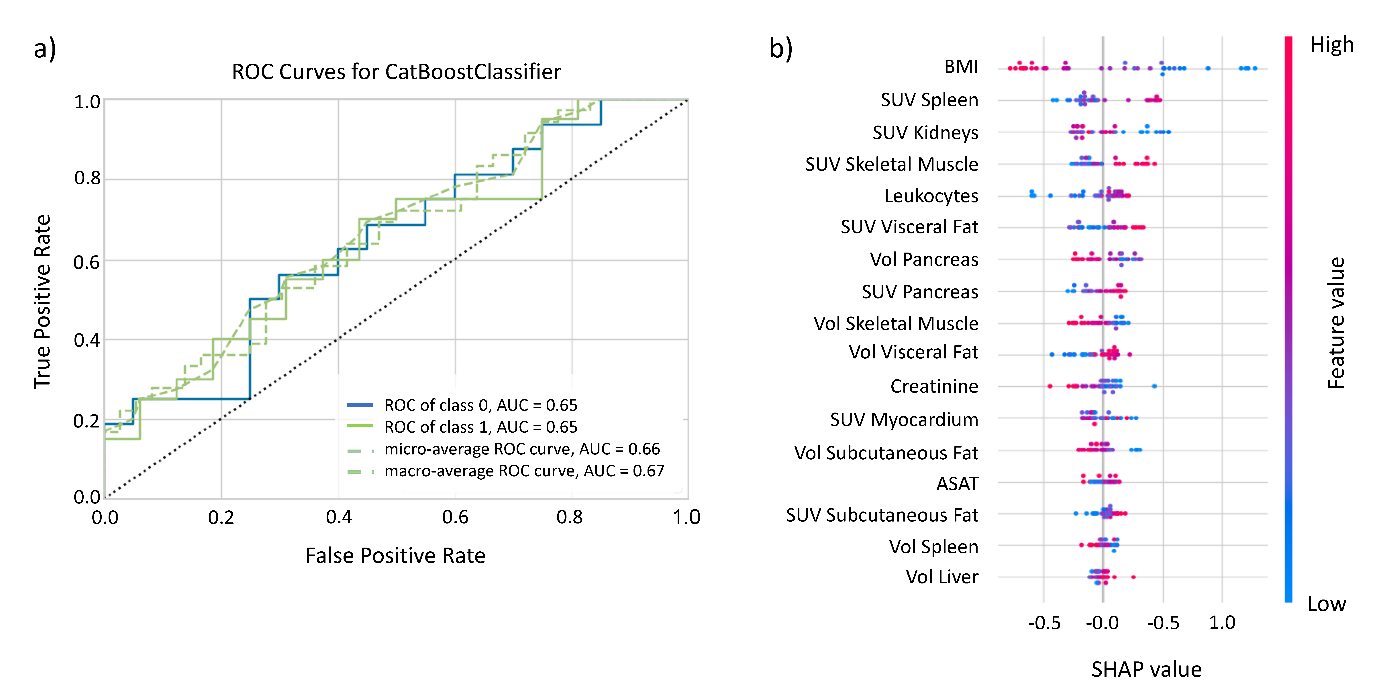


**Fig. S4** CatBoost Classifier ROC curve (a) and SHAP analysis (b) for the binary classification between “No CAC” and “CAC Phenotype” (“Dev CAC” + “CAC”) cohorts. The position of the dots to the left or right in the SHAP plot (b) indicates their influence toward a “No CAC” or “CAC Phenotype” classification, respectively. The colour of the dots indicates the absolute value of each feature: blue for lower and pink for higher values. ASAT = aspartate aminotransferase; BMI = body mass index, SUV = standardized uptake value; Vol = volume.


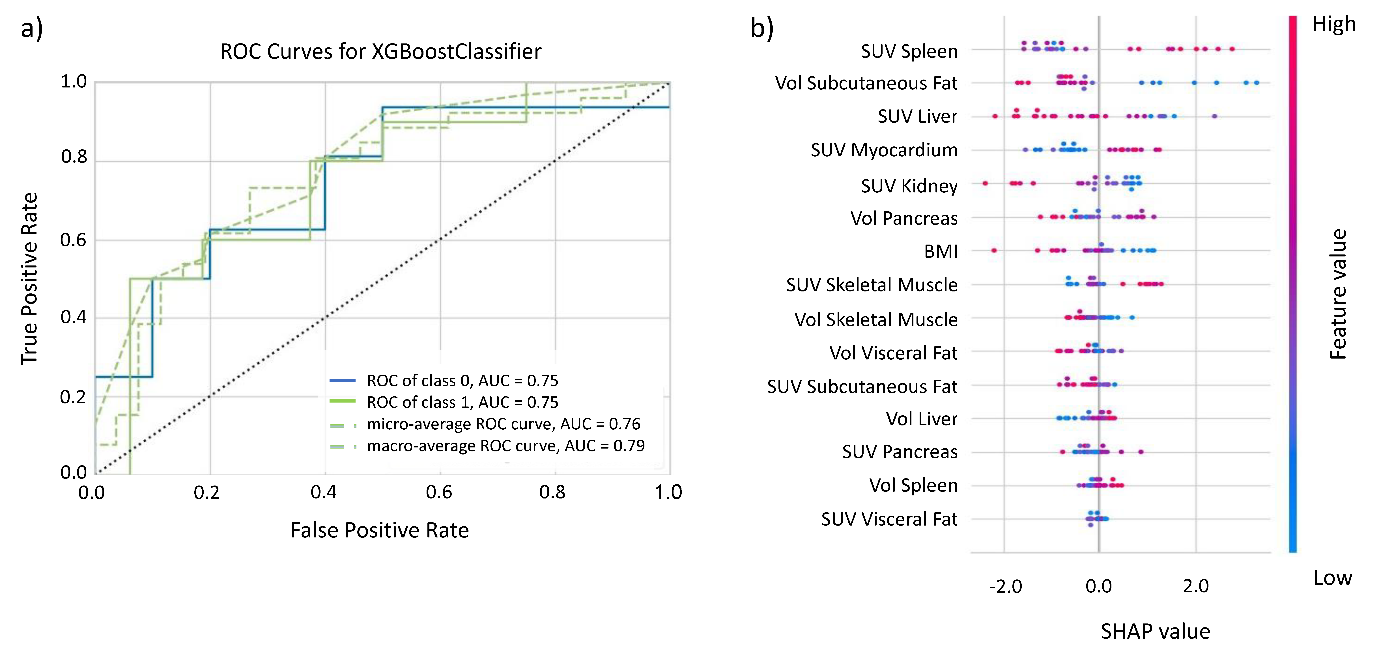


**Fig. S5** XGBoost Classifier ROC curve (a) and SHAP analysis (b) for the binary classification between “No CAC” and “CAC” cohorts, including only BMI and imaging parameters. The position of the dots to the left or right in the SHAP plot (b) indicates their influence toward a “No CAC” or “CAC” classification, respectively. The colour of the dots indicates the absolute value of each feature: blue for lower and pink for higher values. BMI = body mass index, SUV = standardized uptake value; Vol = volume.
