# Supplementary material for: Detection of cancer‐associated cachexia in lung cancer patients using whole‐body [18F]FDG‐PET/CT imaging: A multi‐centre study
Source: J Cachexia Sarcopenia Muscle. 2024 Aug 27;15(6):2375–86. doi: 10.1002/jcsm.13571 (PMC11634466; doi:10.1002/jcsm.13571)
Supplement: Supplementary file 2 — Data S1. Supporting Information [file JCSM-15-2375-s002.docx]

**SUPPLEMENTARY REFERENCES**

[S1. Tárnoki ÁD, Tárnoki DL, Dąbrowska M, Knetki-Wróblewska M, Frille A, Stubbs H, et al. New developments in the imaging of lung cancer. Breathe (Sheff). 2024;20:230176.](https://www.ncbi.nlm.nih.gov/pmc/articles/PMC11003524/)

[S2. Brown LR, Sousa MS, Yule MS, Baracos VE, McMillan DC, Arends J, et al. Body weight and composition endpoints in cancer cachexia clinical trials: Systematic Review 4 of the cachexia endpoints series. J Cachexia Sarcopenia Muscle. 2024;15:816–52.](https://www.ncbi.nlm.nih.gov/pmc/articles/PMC11154800/)

[S3. Anandavadivelan P, Johar A, Lagergren P. The weight loss grading system as a predictor of cancer cachexia in oesophageal cancer survivors. Eur J Clin Nutr. 2022;76:1755–61.](https://pubmed.ncbi.nlm.nih.gov/35982215/)

[S4. Webster JM, Kempen LJAP, Hardy RS, Langen RCJ. Inflammation and Skeletal Muscle Wasting During Cachexia. Front Physiol. 2020;11:597675.](https://www.ncbi.nlm.nih.gov/pmc/articles/PMC7710765/)

[S5. Shen W, Punyanitya M, Wang Z, Gallagher D, St-Onge M-P, Albu J, et al. Total body skeletal muscle and adipose tissue volumes: estimation from a single abdominal cross-sectional image. J Appl Physiol. 2004;97:2333–8.](https://pubmed.ncbi.nlm.nih.gov/15310748/)

[S6. Seelaender MCL, Batista ML. Adipose tissue inflammation and cancer cachexia: the role of steroid hormones. Horm Mol Biol Clin Investig. 2014;17:5–12.](https://pubmed.ncbi.nlm.nih.gov/25372726/)

[S7. Wei L, Wang R, Lin K, Jin X, Li L, Wazir J, et al. Creatine modulates cellular energy metabolism and protects against cancer cachexia-associated muscle wasting. Front Pharmacol. 2022;13:1086662.](https://www.ncbi.nlm.nih.gov/pmc/articles/PMC9767983/)

[S8. Miller J, Wells L, Nwulu U, Currow D, Johnson MJ, Skipworth RJE. Validated screening tools for the assessment of cachexia, sarcopenia, and malnutrition: a systematic review. Am J Clin Nutr. 2018;108:1196–208.](https://pubmed.ncbi.nlm.nih.gov/30541096/)
